# Assessment of Serum Vitamin D and Parathyroid Hormone in Children With Beta Thalassemia Major: A Case-Control Study

**DOI:** 10.7759/cureus.66146

**Published:** 2024-08-04

**Authors:** Rajkumar M Meshram, Manan A Salodkar, Shruti R Yesambare, Somnath M Mohite, Renuka B Gite, Veena S Mugali, Kanchan K Ambatkar, Nandkishor J Bankar, Gulshan R Bandre, Ankit Badge

**Affiliations:** 1 Paediatrics, Government Medical College and Hospital, Nagpur, IND; 2 Microbiology, Jawaharlal Nehru Medical College, Datta Meghe Institute of Higher Education and Research, Wardha, IND; 3 Microbiology, Datta Meghe Medical College, Datta Meghe Institute of Higher Education and Research, Nagpur, IND

**Keywords:** serum ferritin, parathyroid hormone, vitamin d, serum calcium, beta thalassemia major

## Abstract

Background: A defective synthesis of vitamin D contributes to alterations in calcium homeostasis due to chronic endocrinopathies, leading to metabolic bone diseases. This study aimed to ascertain the levels of calcium, vitamin D, and parathyroid hormone (PTH) in children with β-thalassemia.

Methods: In this case-control study, 36 children with major β-thalassemia receiving iron chelation therapy were included. For the control group, 36 cases matched for age and sex were selected. The packed cell volume (PCV) requirements varied among the thalassemic children, with an average PCV requirement of 78.57±49.07. The study was conducted for six months in the Department of Pediatrics at the Government Medical College, Nagpur, India. Serum PTH levels were determined by immunoassay, and serum vitamin D levels were assessed using electrochemiluminescence technique. Additional tests looked at liver function, serum ferritin, calcium, phosphorus, and complete blood count. The student's t-test, Mann-Whitney, and chi-square tests were used for statistical analysis.

Result: In comparison to the control group (10.4±1.21 g/dL), the case group's mean hemoglobin level was considerably lower (5.62±1.9 g/dL) (p<0.001). The mean serum ferritin level in the cases was notably higher (3073±1262.24 ng/mL) compared to the control group's level (58.37±29.67 ng/mL) (p<0.001). A total of 80.6% of cases compared to 5.6% of controls had vitamin D deficiency, and 72.2% of cases compared to 2.8% of controls had PTH deficit, both of which showed statistically significant differences (p<0.001). Significant differences were observed between the case and control groups for the mean levels of total serum calcium (8.51±0.84 mg/dL), vitamin D (15.23±10.07 ng/mL), and PTH (14.66±19.86 pg/mL) (9.13±0.6 mg/dL, p=0.05; 34.94±9.57 ng/mL, p<0.001; 32.08±12.42 pg/mL, p<0.001; respectively).

Conclusion: Growth failure may result from the markedly reduced serum calcium, vitamin D, and PTH levels in children with β-thalassemia. The relevance of treatment approaches is highlighted by the possibility that these anomalies are caused by excessive iron and inadequate nutritional support.

## Introduction

Βeta (β) thalassemia is an autosomal recessive genetic disorder characterized by a quantitative deficiency in the synthesis of β globin chains of hemoglobin, encoded by the hemoglobin (Hb) β gene located on chromosome 11 [1]. Clinically, homozygous β-thalassemia typically presents as thalassemia major or intermedia, whereas heterozygous individuals are known as carriers [2]. Children with β-thalassemia suffer a significant burden of chronic anemia, are dependent on transfusions, and exhibit extramedullary hematopoiesis leading to growth failure, skeletal deformities, transfusion-related infections, iron overload, and complications that affect the cardiac, endocrine, and liver systems, resulting in a lower life expectancy than non-thalassemic children [3].

Around 300,000 to 400,000 children globally are born yearly with inherited hemoglobin diseases, with approximately 80 million carriers of β-thalassemia, representing 1.5% of the world population [1,2,4]. Among these global populations, a large number of β-thalassemia carrier cases originate from the Indian subcontinent, including Bangladesh, Pakistan, and India, and also from neighboring countries such as Malaysia and Thailand [2,5]. The prevalence of β-thalassemia carriers is approximately 4% in the general population and 4.6% in the tribal population of India [5].

In β-thalassemia, defective synthesis of 25-hydroxy vitamin D and hypoparathyroidism alter calcium homeostasis. This impairment is a consequence of iron overload, hormonal deficiencies, marrow expansion, toxicity of chelation therapy, and malnutrition, all of which affect bone metabolism, leading to osteoporosis, rickets, bone fractures, spinal deformities, and nerve compression. However, with adequate and timely transfusion protocols and appropriate chelation therapy, these adverse outcomes can be significantly mitigated, improving overall bone health and reducing the risk of such complications [6-10]. Various studies worldwide have reported lower calcium and vitamin D levels in thalassemic children than in healthy children in diverse age groups [11-14]. This study aimed to ascertain the levels of calcium, vitamin D, and parathyroid hormone (PTH) in children with β-thalassemia.

## Materials and methods

Study design and setting

This case-control study was conducted at the daycare center in the Department of Pediatrics at the Government Medical College, Nagpur (Central India) from September 2023 to February 2024.

Participants

The study included children aged two to 12 years, diagnosed with thalassemia major by high-performance liquid chromatography (HPLC)/Hb electrophoresis. These children were regularly transfused with packed red blood cells (PRBCs) and were receiving regular chelation therapy. Children with other hemoglobinopathies requiring regular transfusion (e.g., sickle cell anemia), those with poor compliance to packed cell transfusion, sick children, children with difficulty in feeding or malnutrition, and those receiving medications including vitamin D, calcium, and phosphorus were excluded. Children on PTH supplementation and those with chronic diseases (cardiac, renal) were also excluded. Age-matched and sex-matched children who attended the pediatric outpatient department (OPD) for minor illnesses, were not suffering from β-thalassemia, and had not received vitamin D or PTH preparations were included in the control group.

Sample size and sampling method

The sample size calculation was conducted using the formula provided by Dupont for matched case-control studies: n=((Z_1−α/2_+Z_1−β_)^2^×(1+(1+ψ)^2^×ϕ)/(ψ−1)^2^×(1−ϕ^2^)), where, α= 0.05 (significance level), β= 0.20 (1 - power, for 80% power), ψ= 2 (odds ratio), ϕ= 0.2 (correlation coefficient for exposure between matched cases and controls), Z_1−α/2_= 1.96 (standard normal deviate for 95% confidence level), Z_1−β_= 0.84 (standard normal deviate for 80% power).

Substituting the values, we got n= ((1.96+0.84)^2^×(1+(1+2)^2^×0.2)/(2−1)^2^×(1−0.2^2^)) or n= ((2.8)^2^×(1+9×0.2)/(1)^2^×(1−0.04)) or n= (7.84×(1+1.8)/1×0.96) or n= (7.84×2.8/0.96) or n≈ 22.87.

Rounding up to the nearest integer, the required sample size per group was approximately 23. However, in the present study, 36 cases and 36 controls were enrolled using a consecutive sampling method, based on the availability of patients within the data collection period.

Ethical considerations

All necessary information regarding the study was explained to parents/guardians in the vernacular language before enrolling the participants, and informed written consent/assent was obtained from those willing to participate. The study was approved by the Institutional Ethics Committee (No.2529/EC/Pharmac/GMC/NGP dated 25/10/2021).

Data collection

Demographic details including age, sex, residence, socio-economic status, status of parents/siblings, age at first transfusion, frequency of transfusion, iron chelation therapy, duration of chelator therapy, and immunization status were collected.

Laboratory investigations

Biochemical and Hematological Analysis

Five milliliters of venous blood were drawn under aseptic settings. An automated hematology analyzer was used to analyze the whole blood count after milliliters of whole blood were collected and placed in an ethylenediamine tetraacetic acid (EDTA) vacutainer. After clotting at room temperature for 30 minutes, four milliliters of blood were placed in a simple test tube and centrifuged at 1000× g for 15 minutes. After being separated, the serum was aliquoted and kept at less than -20°C until needed again.

Assays

Serum ferritin: It was estimated by enzyme-linked immunosorbent assay (ELISA) with a reference range of 20-280 ng/mL for men and 10-140 ng/mL for females.

Serum calcium, phosphorus, magnesium, and liver enzymes: They were estimated using a Beckman Coulter (AU5800; Beckman Coulter Diagnostics, Brea, California, United States) automated analyzer, with appropriate kits and chemical principles.

Serum vitamin D: It was estimated using electrochemiluminescence technology on a fully automatic COBAS e411 analyzer (Roche Holding AG, Basel, Switzerland). Serum vitamin D levels less than 20 ng/mL were considered deficient.

Serum PTH: It was estimated by immunoassay on a COBAS e411 analyzer with a reference range of 15-51 pg/mL. Serum PTH levels less than 15 pg/mL were considered deficient.

Statistical analysis

Data was entered into a Microsoft Excel spreadsheet (Microsoft Corporation, Redmond, Washington, United States) and analyzed using Epi Info for Windows, version 7.2 (CDC, Atlanta, Georgia, United States. Frequency, mean, and standard deviation were calculated. The Student’s t-test, Mann-Whitney test, and chi-square test were used for comparing cases and controls as appropriate. A p-value of less than 0.05 was considered statistically significant.

## Results

This study included 36 children with major β-thalassemia or transfusion dependence (case group) and 36 age- and sex-matched children with minor illnesses such as upper respiratory tract infections (control group). The age range for both groups was two to 12 years. There were no significant differences between the groups in terms of gender, residence, socioeconomic status, and immunization status, except for pneumococcal vaccination, which was significantly higher in the case group (33.3%) compared to the control group (5.6%) (p=0.003).

Demographic characteristics

The demographic characteristics of the study participants, including age, gender, residence, socioeconomic status, immunization status, and anthropometric measurements are presented in Table 1. Gender, location of residence, and socioeconomic position did not significantly differ between the cases and controls. On the other hand, the case group's weight and height were considerably lower than those of the control group.

**Table 1:** Demographic characteristics of study participants SD: standard deviation; NIP: National Immunization Program; kg: kilogram; cm: centimeter; t: T-test; χ: chi

Characteristic	Cases (n=36)	Control group (n=36)	p-value	Statistic (value)
Age (years) (mean±SD)	6.59±2.70	6.56±2.86	0.9697	t = -0.046
< five years (%)	8 (22.2%)	10 (27.8%)	0.586	χ² = 0.297
> five years (%)	28 (77.8%)	26 (72.2%)	0.586	
Gender				
Male (%)	22 (61.1%)	23 (63.9%)	0.808	χ² = 0.059
Female (%)	14 (38.9%)	13 (36.1%)		
Residence				
Rural (%)	28 (77.8%)	25 (69.4%)	0.422	χ² = 0.644
Urban (%)	8 (22.2%)	11 (30.6%)		
Socio-economic Status				
Lower (%)	30 (83.3%)	27 (75%)	0.384	χ² = 0.741
Middle (%)	6 (16.7%)	9 (25%)		
Immunization status				
Complete as per NIP (%)	34 (94.4%)	36 (100%)	0.493	χ² = 2.045
Pneumococcal vaccination (%)	12 (33.3%)	2 (5.6%)	0.003	χ² = 8.693
Anthropometry (mean±SD)				
Weight (kg)	11.8±2.66	17.48±6.25	<0.0001	t =5.017
Height (cm)	90.5±12.26	110.51±19.25	0.023	t =5.261

Hematological and biochemical parameters 

The hematological and biochemical parameters of the study participants are described in Table 2. In comparison to the control group, the case group's mean hemoglobin level was considerably lower. Likewise, there was a significant difference in the mean serum ferritin level between the patients and controls. Additionally, the case group's platelet and white blood cell counts were significantly lower. Moreover, the case group had significantly lower cholesterol levels and significantly higher levels of serum alanine aminotransaminase (ALT), triglycerides, and alkaline phosphatase.

**Table 2:** Hematological and biochemical parameters in cases and controls SD: standard deviation; Hb: hemoglobin; g: grams; dL: deciliter; WBCs- white blood cells; μL: microliter; ng: nanograms; ALT: alanine aminotransaminase, AST: aspartate aminotransferase; U: units; L: liter; KAU: King-Armstrong unit; mg: milligrams; Meq: milliequivalents

Parameter	Cases (Mean±SD)	Control group (Mean±SD)	p-value	t-statistic
Hb (g/dL)	5.62±1.9	10.4±1.21	<0.0001	12.732
WBCs (10^3^/μL)	7.9±4.7	9.5±3.5	0.004	1.638
Platelets (10^3^/μL)	280.58±164.22	329.72±102.58	0.0357	1.523
Ferritin (ng/dL)	3073±1262.24	58.37±29.67	<0.0001	-14.326
ALT (U/L)	43.25±21.42	33.55±12.98	0.0284	-2.324
AST (U/L)	36.77±19.97	33.36±12.42	0.7779	-0.87
Alkaline phosphatase (KAU/L)	199.5±127.86	150.27±60.16	0.0403	-2.09
Cholesterol (mg/dL)	95.85±32.97	129.88±30.19	<0.0001	4.567
Triglycerides (mg/dL)	155.11±57.66	100.44±27.79	<0.0001	-5.125
Blood urea (mg/dL)	21.94±7.62	22.61±8.92	0.7344	0.343
Creatinine (mg/dL)	0.34±0.1	0.46±0.17	0.0009	3.651
Serum sodium (Meq/L)	133.83±3.90	136.86±4.95	0.0053	2.885
Serum potassium (Meq/L)	4.14±0.62	4.25±0.32	0.3215	0.946

Comparison of calcium, phosphorus, PTH, and vitamin D levels

The levels of calcium, phosphorus, magnesium, vitamin D, and PTH in the study participants are described in Table 3. The mean levels of total serum calcium, vitamin D, and PTH levels were significantly lower in the cases compared to the controls. Conversely, the mean serum phosphorus level was significantly higher in the case group compared to the control group.

**Table 3:** Comparison of calcium, phosphorus, parathyroid hormone, and vitamin D levels between cases and controls SD: standard deviation; mg: milligrams; dL: deciliter; ng: nanograms; mL: milliliter; PTH: parathyroid hormone; pg: picograms

Variable	Cases (mean±SD)	Control group (mean±SD)	p-value	t-statistic
Calcium (mg/dL)	8.51±0.84	9.13±0.6	0.05	3.604
Phosphorus (mg/dL)	5.04±2.10	4.15±0.72	0.0201	-2.405
Magnesium (mg/dL)	1.70±0.56	2.00±0.346	0.0521	2.734
Vitamin D (ng/mL)	15.23±10.07	34.94±9.57	<0.001	8.513
PTH (pg/mL)	14.66±19.86	32.08±12.42	<0.0001	4.462

Vitamin D and PTH deficiency

The prevalence of vitamin D and PTH deficiencies in study participants is described in Table 4. Vitamin D deficiency was significantly more prevalent in the case group compared to the control group. Similarly, PTH deficiency was significantly more prevalent in the case group compared to the control group. 

**Table 4:** Prevalence of vitamin D and PTH deficiencies in cases and controls ng: nanograms; mL: milliliter; pg: picograms; PTH: parathyroid hormone

Variable	Cases (n=36) %	Control group (n=36) %	p-value	Chi-squared
Vitamin D (ng/mL) <20	29 (80.6%)	2 (5.6%)	<0.0001	40.713
Vitamin D (ng/mL) >20	7 (19.4%)	34 (94.4)	<0.0001	40.713
PTH (pg/mL) <15	26 (72.2%)	1 (2.8%)	<0.0001	36.476
PTH (pg/mL) >15	10 (27.8%)	35 (97.2%)	<0.0001	36.476

## Discussion

This study demonstrates that children with major β-thalassemia have significant deficiencies in serum calcium, vitamin D, and PTH compared to healthy controls. These findings emphasize the significant impact of β-thalassemia on bone metabolism and overall growth, underscoring the need for targeted therapeutic interventions.

Although mortality has improved because of the availability of transfusion, chelation therapy, and hematopoietic stem cell transplantation facilities, children with thalassemia are prone to numerous complications from the disease itself or the consequences of treatment; like cardiac arrhythmia/failure, multiple endocrinopathies, growth failure, and metabolic bone diseases [2]. Although mortality in thalassemia has decreased substantially in Western countries, Dhanya et al. reported that patients with transfusion-transmitted infections had a 3.4 times higher risk of death [3]. Growth failure in thalassemic children is multifactorial, including chronic hypoxia due to chronic anemia, poor feeding, endocrinopathies, fractures, chelation toxicity, and liver dysfunction. Similar to the observations of various authors [8,9,11,15], we observed weight and height were significantly lower in the thalassemic group than in the control group.

In this study, all of the cases of β-thalassemia had pallor, with 86.1% of cases requiring monthly transfusion. The mean age of the first transfusion was 11.77±10.89 months, and the mean transfusion frequency was 78.63±48.91. Thirty thalassemic children had received chelation therapy and all were on oral deferasiorx. The mean duration of therapy was 3.51±2.84 years. Shah et al. revealed that the mean age of blood transfusion was 13.5±6 months and the mean age of starting iron chelation therapy was 4.5±3.2 years in their study [16]. Splenomegaly was noted in 61.1% while splenectomy was done in 25% of cases. This agrees with the findings of Abdelmotaleb et al. who reported that 96% of cases had pallor, nine cases had undergone splenectomy, and 36 cases had splenomegaly [11]. Similarly, Behairy et al. found moderate to severe pallor and hepatomegaly in all cases, while splenomegaly in 75.7% with splenectomy performed in 14.3 % of cases [17].

In the thalassemic group, ALT, alkaline phosphatase, and triglycerides were significantly raised while cholesterol was significantly lower compared to the control group; this could be due to the progressive accumulation of iron and liver parenchymal hemosiderosis. Dyslipidemia in thalassemic patients is caused by several factors, such as plasma dilution due to anemia, an increase in erythropoietic activities followed by an increase in cholesterol uptake by macrophages and histocytes, hormonal disturbances, and a decrease in extrahepatic lipolysis activity. Various researchers have observed elevated liver enzymes and dyslipidemia [18-20].

In the current study, the mean serum level of 25-OH-vitamin D (15.23 ±10.07ng/ml) was significantly much lower in the thalassemic group compared to controls (39.94±9.57ng/ml); 29/36 cases (80.6%) had vitamin D deficiency compared to 2/36(5.6%) in the control group. Ahmed et al. from India and Sultan et al. from Pakistan reported significantly low standard reference values of vitamin D levels in β-thalassemia children [21,22]. Similarly to our observation, various authors reported a significantly lower level of 25-OH-vitamin D level in patients with β-thalassemia than control [11,14,15,22,23]. Handattu et al., Abdelmotaleb et al., and Bulgurcu et al. reported 60%, 49%, and 79%, respectively, cases of β-thalassemia with deficient Vitamin D levels [9-11]. In spite of good sun exposure and routine prescription of vitamin D prescription, such a low level of vitamin D in thalassemic children may be due to endocrinopathies, liver siderosis, dark skin, and poor nutrition.

In the present study, 72.2% of thalassemic children had a low level of PTH compared to 2.8% in control and this difference was statistically significant. Similarly, the mean serum PTH level was significantly lower in the case group (14.66±19.86 pg/ml) than in controls (32.08±12.42pg/ml). Hypoparathyroidism is significantly associated with older age, the mean received blood transfusion, total transfused blood per year, splenomegaly, hepatomegaly, and chelation regimen, while splenectomy is an independent risk factor for low PTH [24]. The reported incidence of hypoparathyroidism ranges from 2-38% in thalassemic children [9,10,24-26]. Such a high incidence of hypoparathyroidism in our study could be because most of our thalassemic children were older, received monthly blood transfusions, and 25% of cases had splenectomy performed. In contrast to our findings, Agarwal et al. reported a high mean PTH level in thalassemic children (64.35±16.01pg/ml) compared to control (42.61±13.75pg/ml) [13].

In this study, the total mean serum calcium and magnesium levels were significantly lower in thalassemic children compared to the control, while the total mean phosphorus level was significantly higher in the case group than in the control group. Hypocalcemia and hyperphosphatemia are explained by iron overload, liver hemosiderosis, chelation therapy, and hypoparathyroidism. Bulgurcu et al. and Abdelmotaleb et al. reported a low mean serum calcium and high phosphorus level in thalassemic children, but a few authors did not find significant hyperphosphatemia in thalassemic children and control [15,23].

Limitation

In this study, there are several limitations to consider. The sample size was relatively small, and the study was conducted at a single center, which may limit the generalizability of the findings. Future research with larger, multicenter cohorts and longitudinal designs is needed to confirm these findings and explore the underlying mechanisms in more detail.

## Conclusions

Thalassemic children have significant growth failure, lower total mean serum calcium, vitamin D, and PTH, high phosphorus levels, ALT and alkaline phosphatase, and dyslipidemia, as compared to controls, which signifies the importance of targeted therapeutic interventions to address this deficiency. Higher liver enzymes and phosphorus levels are likely due to iron overload. Regular monitoring and early intervention are essential to prevent metabolic bone diseases and improve overall health outcomes. Nutritional support and appropriate chelation therapy to manage iron overload are required. A multidisciplinary approach involving pediatricians, endocrinologists, nutritionists, and hematologists is necessary for these children.

## References

[1] Origa R (2017). β-thalassemia. Genet Med.

[2] Yousuf R, Akter S, Wasek SM, Sinha S, Ahmad R, Haque M (2022). Thalassemia: a review of the challenges to the families and caregivers. Cureus.

[3] Dhanya R, Sedai A, Ankita K (2020). Life expectancy and risk factors for early death in patients with severe thalassemia syndromes in South India. Blood Adv.

[4] De Sanctis V, Kattamis C, Canatan D (2017). β-thalassemia distribution in the old world: an ancient disease seen from a historical standpoint. Mediterr J Hematol Infect Dis.

[5] Sumedha D, Anita K (2023). Prevalence of beta thalassemia carriers in India: a systematic review and meta-analysis. J Community Genet.

[6] Soliman A, De Sanctis V, Yassin M (2013). Vitamin d status in thalassemia major: an update. Mediterr J Hematol Infect Dis.

[7] Mahmoud RA, Khodeary A, Farhan MS (2021). Detection of endocrine disorders in young children with multi-transfused thalassemia major. Ital J Pediatr.

[8] Arab-Zozani M, Kheyrandish S, Rastgar A, Miri-Moghaddam E (2021). A systematic review and meta-analysis of stature growth complications in β-thalassemia major patients. Ann Glob Health.

[9] Bulgurcu SC, Canbolat Ayhan A, Emeksiz HC, Ovali F (2021). Assessment of the nutritional status, bone mineralization, and anthropometrics of children with thalassemia major. Medeni Med J.

[10] Handattu K, Aroor S, Kini P, Ramesh Bhat Y, Shivakumar G, Shastry P, Shetty S (2022). Metabolic bone disease in children with transfusion-dependent thalassemia. Indian Pediatr.

[11] Abdelmotaleb GS, Behairy OG, El Azim KE, El-Hassib DM, Hemeda TM (2021). Assessment of serum vitamin D levels in Egyptian children with beta-thalassemia major. Egypt Pediatr Assoc Gaz.

[12] Ridha NR, Gautama J, Ganda IJ (2022). Analysis of vitamin D levels in children with thalassemia beta. Int J Health Sci Med Res.

[13] Agrawal A, Garg M, Singh J, Mathur P, Khan K (2016). A comparative study of 25 hydroxy vitamin D levels in patients of thalassemia and healthy children. Pediatr Rev Int J Pediatr Res.

[14] Gombar S, Parihar K, Choudhary M (2018). Comparative study of serum ferritin and vitamin D in thalassemia patients with healthy controls. Int J Res Med Sci.

[15] Bashir A, Habib K, Lail A, Shah MA, Memon NA, Dahri ZA (2023). Status of vitamin D and evaluation of growth parameters seen in the children suffering from thalassemia major. Pak J Med Health Sci.

[16] Shah B, Gosai D, Shah H (2017). Study of vitamin D status and bone age in children with thalassemia major. Int J Med Sci Clin Invent.

[17] Behairy OG, Abd Almonaem ER, Abed NT (2017). Role of serum cystatin-C and beta-2 microglobulin as early markers of renal dysfunction in children with beta thalassemia major. Int J Nephrol Renovasc Dis.

[18] Nasir C, Rosdiana N, Lubis AD (2018). Correlation between 25-hydroxyvitamin D and lipid profile among children with beta thalassemia major. Open Access Maced J Med Sci.

[19] Salama KM, Ibrahim OM, Kaddah AM, Boseila S, Ismail LA, Hamid MM (2015). Liver enzymes in children with beta-thalassemia major: correlation with iron overload and viral hepatitis. Open Access Maced J Med Sci.

[20] Darvishi-Khezri H, Karami H, Naderisorki M, Zahedi M, Razavi A, Kosaryan M, Aliasgharian A (2020). Moderate to severe liver siderosis and raised AST are independent risk factors for vitamin D insufficiency in β-thalassemia patients. Sci Rep.

[21] Ahmed Z, Pushpanjali Pushpanjali, Kausar MS, Sinha D (2019). Study of serum calcium, phosphorus and vitamin D status in multitransfused β-thalassemia major children and adolescents of Jharkhand, India. Int J Contemp Pediatr.

[22] Sultan S, Irfan SM, Ahmed SI (2016). Biochemical markers of bone turnover in patients with β -thalassemia major: a single center study from Southern Pakistan. Adv Hematol.

[23] Fahim FM, Saad K, Askar EA, Eldin EN, Thabet AF (2013). Growth parameters and vitamin D status in children with thalassemia major in upper Egypt. Int J Hematol Oncol Stem Cell Res.

[24] Bazi A, Harati H, Khosravi-Bonjar A, Rakhshani E, Delaramnasab M (2018). Hypothyroidism and hypoparathyroidism in thalassemia major patients: a study in Sistan and Baluchestan Province, Iran. Int J Endocrinol Metab.

[25] Tangngam H, Mahachoklertwattana P, Poomthavorn P, Chuansumrit A, Sirachainan N, Chailurkit L, Khlairit P (2018). Under-recognized hypoparathyroidism in thalassemia. J Clin Res Pediatr Endocrinol.

[26] De Sanctis V, Soliman AT, Canatan D (2018). An ICET- a survey on hypoparathyroidism in patients with thalassaemia major and intermedia: a preliminary report. Acta Biomed.

